# Gender differences in self-rated health among older adults in the Chinese workforce

**DOI:** 10.3389/fpubh.2024.1450045

**Published:** 2024-12-06

**Authors:** Wenyu Li, Zhijie Xu, Wenjie Tang

**Affiliations:** ^1^School of Marxism, Capital Normal University, Beijing, China; ^2^School of Economics and Management, North China Electric Power University, Beijing, China; ^3^Capital Normal University, Beijing, China

**Keywords:** self-rated health, gender differences, older adult workforce, economic factors, lifestyle habits

## Abstract

**Introduction:**

This study investigates gender-based disparities in self-rated health among older individuals in the Chinese workforce, utilizing data from the China Health and Retirement Longitudinal Study (CHARLS). Understanding these health gaps is crucial for crafting effective health policies and interventions in light of the rapidly aging population.

**Materials and methods:**

Data from the 2020 CHARLS survey, comprising Chinese individuals aged 45 and above, were analyzed, focusing on older adults actively employed. Descriptive statistics and regression analyses examined gender discrepancies in self-rated health, considering diverse sociodemographic, economic, and health-related factors.

**Results:**

Gender disparities in demographics, work environments, and self-rated health were observable among male and female participants. Older males tended to have higher rates of smoking and alcohol consumption, coupled with lower incomes. In contrast, females exhibited healthier behaviors influenced by access to healthcare and lifestyle modifications. For males, economic stability and moderate alcohol use positively influenced self-rated health, while females benefited from healthcare coverage and healthy lifestyle choices. Tailored gender-specific health interventions should prioritize these unique factors to enhance overall well-being.

**Discussion:**

Discussions highlighted the impact of demographic variables, including age, marital status, social security, and employment conditions, on self-rated health. The study emphasized the crucial role of marital relationships in the health outcomes of older adult workers.

**Conclusion:**

This study underscores the pivotal role of gender in self-rated health variations and provides essential insights for targeted interventions. By considering both quantitative and qualitative determinants of well-being, focused health policies can effectively address the health and well-being of aging populations, especially older adult workers.

## Introduction

Population aging is a significant global challenge. According to the data from the United Nations, by 2050, one out of every six people in the world will be over 65 years old, which is three times higher than the proportion in 1990 (1). However, productive labour, instead of being a means of subjugating men, should become a means of their emancipation, by offering each individual the opportunity to develop all his faculties, physical and mental, in all directions and exercise them to the full—in which, therefore, productive labour should become a pleasure instead of being a burden (2). Instead of being seen as burdensome or irrelevant individuals, the social value of older adult workforce should be recognized. To achieve this, a thorough investigation of the health status of them is necessary. By addressing their specific health needs and challenges, policymakers can personalize interventions to promote their well-being and maximize their contribution to society, while also enabling the realization of their individual value in old age.

Previous studies (3–5) have not reached a consensus regarding gender differences in self-rated health, and research on the older adult workforce has also indicated the presence of gender discrimination in the labor market. Self-rated health serves as a vital marker, reflecting objective health conditions and correlating with long-term cardiovascular health. Research indicates that self-reporting health status is a critical indicator of cardiovascular health (6) and is closely associated with long-term physical morbidity and mortality (7). Based on data from China, this study aims to reveal gender disparities in self-rated health among the older adult workforce through empirical research. The findings will provide evidence-based interventions to enhance the health and well-being of older adult workforce, promote gender equality, and serve as a reference for policymakers seeking to fully utilize the social value of the older adults.

## Literature context

Gender differences in self-rated health have been a topic of extensive research. However, the existing literature has yielded inconsistent findings regarding gender disparities in self-rated health. Some studies suggest no gender differences in self-rated health. Arber et al. (8) found that while older women had higher levels of functional impairment compared to men, there were no gender differences in self-rated health. Conversely, a study on self-rated health during adolescence indicated higher levels of self-rated health among females. Ostberg et al. (9) observed that adolescent girls scored higher on behavioral indicators, displayed greater interest in oral health, and perceived their oral health condition to be better than boys. D’Ambrosio et al. (10) also noted differences in self-regulatory behavioral patterns between males and females. Other research suggests that although women have longer lifespans, their self-rated health tends to be poorer than that of men. Knodel et al. (11) reported that despite higher survival rates among women, their self-rated health tends to be worse than that of men, with more health symptoms and physical functioning issues.

Scholars have demonstrated that gender differences in self-rated health diminish over time. Zajacova et al. (12) found that women generally rated their health lower than men, but this disparity decreased in old age and disappeared after accounting for socioeconomic and health covariates. Other researchers have examined gender differences in self-rated health and other influencing factors among the aging population. Chen et al. (13) investigated the gender differences in the impact of parental bereavement on health outcomes, including self-rated health, among middle-aged and older adults. Sharma et al. (14) delved into the potential mechanisms of age and gender differences in self-rated risk tolerance among the aging population, focusing on cognitive functioning and emphasizing the importance of understanding cognitive function in mitigating low risk tolerance in older adults. Lastly, Qi et al. (15) emphasized the prevalence and risk factors of social frailty in older adult individuals with cardiovascular diseases in China, highlighting an understudied aspect of frailty within this population.

Previous studies in the context of older adult workforce have primarily focused on the economic benefits or have regarded older individuals as a societal burden or recipients of care. Johnson (16) argued that, apart from the interest in the impact of pay-as-you-go pension systems on future tax burdens, economists seem to have generally overlooked the issues of older individuals and population aging. Dhar et al. (17) highlighted that population aging is rapidly becoming a significant socio-economic concern in India, with the rising dependency ratio imposing economic burdens on families. Conrad et al. (18) emphasized that population aging in Japan not only increases the burden on the younger generation but also depletes the resources necessary for driving robust economic growth.

While some studies have mentioned gender differences in the older adult workforce, there is often an emphasis on older women as wives and family members when they become the focus of research. Marenzi et al. (19) analyzed the empirical relationship between female labor market participation and intergenerational family ties using data from the 2000 Bank of Italy’s Survey on Household Income and Wealth (SHIW). Dempse et al. (20) noted that in Australia, retired men tend to assist rather than share the majority of traditional female household chores, with tasks requiring significantly less time and often of lower quality compared to the work of domestic helpers, while not significantly reducing their personal autonomy. Barone et al. (21) investigated whether and how the inflow of female immigrants specializing in household production affects the labor supply of Italian women.

Women are often studied as a specific labor force, namely caregivers. Finch et al. (22) found that care in residential institutions heavily relies on low-wage female labor. Ogawa et al. (23) examined the impact of providing care for older adult parents on the labor force participation of middle-aged women in Japan. The results indicated that regardless of the level of disability among older adult parents, the probability of daughters or daughters-in-law being employed is approximately 75%.

In summary, previous research has not yet reached a consensus on gender differences in self-rated health among the older adult workforce, and these findings have shown variation over time. Older adult workers are often viewed as a societal burden or recipients of care, with older adult women workers typically classified as caregivers, forming a distinct occupational group. Consequently, past studies have exhibited a pessimistic tendency, and older adult female workers also face tendencies of occupational discrimination.

## Materials and methods

### Source of data

This study utilized data from the China Health and Retirement Longitudinal Study (CHARLS).^1^ CHARLS is a nationally representative, interdisciplinary public database aimed at understanding the health and well-being of individuals aged 45 and above in China (24). The present study utilized the 2020 wave of the CHARLS national baseline survey, covering 19,351 individuals and over 10,000 households. The selection of the CHARLS baseline survey was motivated by several factors: it provides detailed data on individuals’ socioeconomic status, health conditions, and work situations, which are essential for exploring the health and well-being of China’s older adult labor force. Moreover, this dataset offers an opportunity to investigate the impacts of delayed retirement and gender equality on the health and well-being of the older adult workforce force. Since this study was a secondary analysis of CHARLS data, we did not require a separate ethical approval.

### Model

This study utilized Stata 17.0 statistical software to conduct descriptive analysis and provide a detailed report on the differences in demographic characteristics, health conditions, and lifestyles between older adult individuals who are engaged in labor and those who are not. Additionally, chi-square tests are used to analyze the intergroup differences in various variables between these two groups. Logistic regression analysis is conducted to explore the correlations between the older adult workforce and various factors. All significant differences (*p* < 0.05) are included in the regression model. Results with a *p*-value less than 0.05 are considered statistically significant.

### Dependent variables

The key variable of this study is gender, but specific selection criteria is required for the study population. Firstly, the selection of age range is crucial as there is a lack of clear age definition for the older adult workforce in research. Previous research (25) has suggested research should more broadly include employees aged 60 and above with the acceleration of population aging, so, we selected the older adult population aged 60 and above. Additionally, we determined individuals’ retirement status by using the questionnaire item “Have you already retired?” (FH001), and we utilized the Xworking indicator in the questionnaire to determine whether individuals in the target age group were still engaged in labor activities. Based on the above considerations, the study population is defined as individuals aged 60 and above who are actively participating in labor activities.

**Figure 1:**
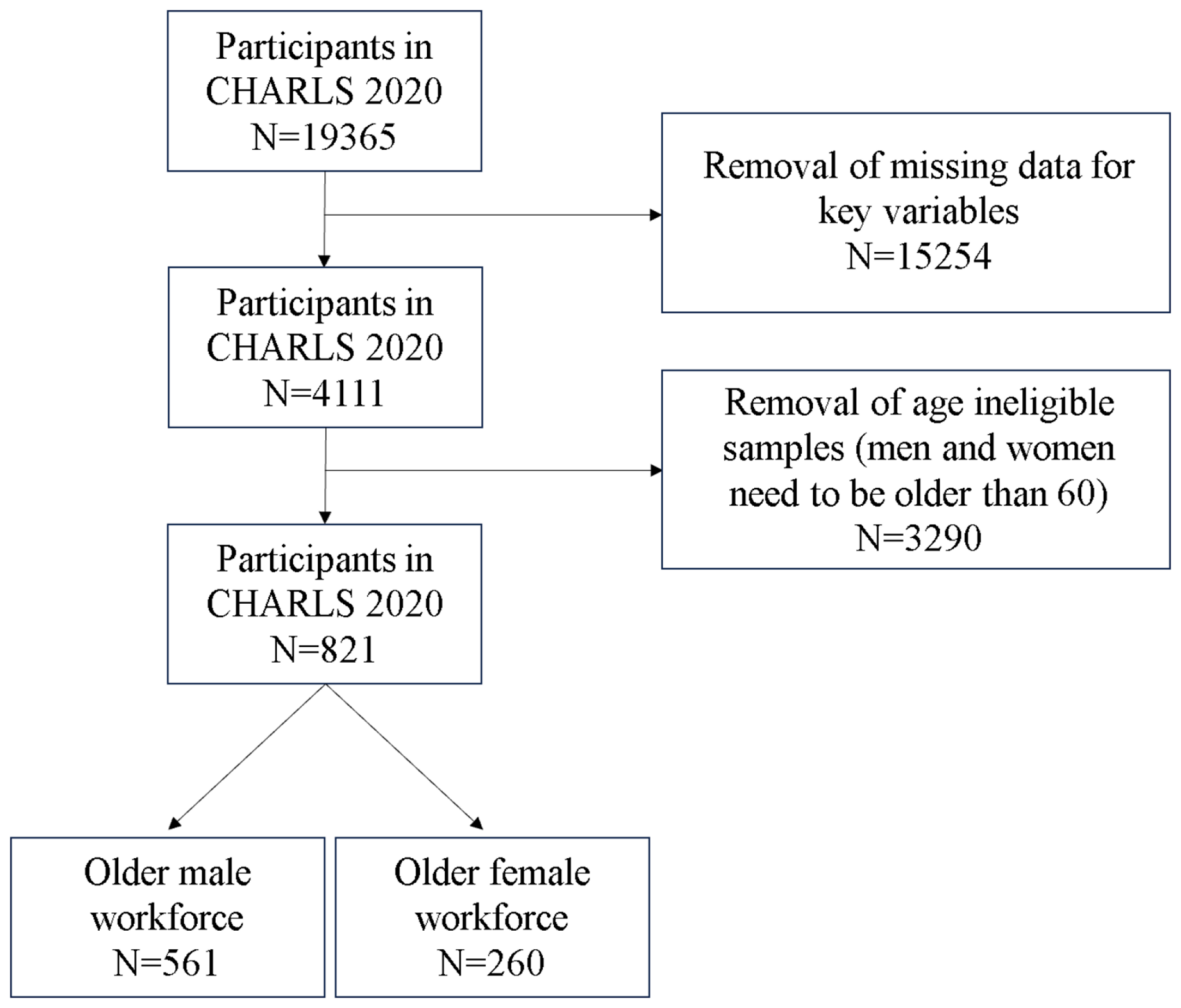
Data filtering flowchart.

In this study, the explanatory variables are analyzed from several aspects, including demographic information, social security, employment status, and individual habits. To provide a clearer representation of the data processing flow, we present a data filtering flowchart (as shown in Figure 1). Demographic information encompasses age, Residence Location, Household registration type, and marital status. Social security includes pension insurance and medical insurance. Employment status includes type of employer, number of days worked per week in the past year, ways of receiving salary, and income level. Lifestyle habits include smoking and drinking habits, duration of sleep, and dietary intake. Detailed variable assignments are given in the Supplementary material S1.

## Results

### Statistical analysis

**Table 1:** 

Items	Male		Female		*p*-value
Age	64.4385		64.70385		<0.001a
Residence location					0.3143^b^
Urban or town center	123	21.93%	46	17.69%	
Urban–rural or town-village junction area	80	14.26%	35	13.46%	
Rural area	358	63.81%	179	68.85%	
Type of household registration					0.0551^b^
Agricultural household registration	455	81.11%	224	86.15%	
Non-agricultural household registration	56	9.98%	13	5.00%	
Non-resident household registration	50	8.91%	23	8.85%	
Marital status					<0.001^b^
Without spouse	49	8.73%	60	23.08%	
With spouse	512	91.27%	200	76.92%	
Pension insurance					
No	0	0	0	0	
Yes	561	100%	260	100%	
Medical insurance					0.424^b^
No	18	3.21%	12	4.62%	
Yes	543	96.79%	248	95.38%	
Information of employment					0.005^b^
Government	73	13.01%	20	7.69%	
Public institution	26	4.63%	7	2.69%	
Non-profit organization (NPO)	4	0.71%	2	0.77%	
Enterprise	164	29.23%	62	23.85%	
Self-employed household	200	35.65%	98	37.69%	
Farming household	74	13.19%	59	22.69%	
Resident household	20	3.57%	12	4.62%	
Whether you are a manager or not					<0.001^b^
Non-supervisory position	482	85.92%	253	96.93%	
Management position	79	14.08%	8	3.07%	
Number of days worked per week in the past year	5.308378		5.161538		<0.001^a^
Salary					0.0945^b^
Regular wage	490	87.34%	215	82.69%	
Performance-based wage	71	12.66%	45	17.31%	
Income					<0.001^b^
Low wage level	296	52.76%	196	75.38%	
Medium-low wage level	140	24.96%	34	13.08%	
Medium wage level	86	15.33%	25	9.62%	
Medium-high wage level	35	6.24%	5	1.92%	
High wage level	4	0.71%	0	0	
Cigarettes					<0.001^b^
Non-smoker	224	39.93%	247	95%	
Smoker	337	60.07%	13	5%	
Alcohol					<0.001^b^
Non-drinker	202	36.01%	221	85%	
Drinker	359	63.99%	39	15%	
Changes in sleep duration					0.3536^b^
Decreased significantly	18	3.21%	11	4.23%	
Small decrease	33	5.88%	20	7.69%	
No change	456	81.28%	213	81.92%	
Small increase	26	4.63%	6	2.31%	
Significant increase	28	4.99%	10	3.85%	
Changes in food-intake					0.3655^b^
Decreased significantly	22	3.92%	9	3.46%	
Small decrease	36	6.42%	11	4.23%	
No change	493	87.88%	232	89.23%	
Small increase	5	0.89%	2	0.77%	
Significant increase	5	0.89%	6	2.31%	
Self-rated health					<0.001^b^
Not at all satisfied	10	1.78%	15	5.79%	
Not very satisfied	18	3.21%	24	9.27%	
Quite satisfied	323	57.58%	123	47.49%	
Very satisfied	188	33.51%	87	33.59%	
Extremely satisfied	22	3.92%	10	3.86%	

In this context, “a” represents the *t*-test, and “b” represents the chi-square test, where significance is defined as *p* > 0.05. Due to space constraints, the figures and tables presented here show the results after filtering for significant indicators. Please refer to the appendix for further details.

### Analysis of the baseline statistical results

In this section, we conducted a comparative analysis of the demographics and working conditions between male and female respondents. Significant differences were observed: the average age of males was 64.44 years, while females averaged 64.70 years. Married males significantly outweighed females (91.27% vs. 76.92%). Notably, males were more likely to be self-employed or work in companies, and a higher proportion of males occupied managerial positions (14.08% vs. 3.07% for females). Income disparities were also evident, with 52.76% of males in the low-income bracket compared to 75.38% of females. Health habits revealed marked gender differences: 60.07% of males smoked, vastly exceeding the 5% of females who reported smoking. Similarly, alcohol consumption was much higher among males (63.99%) than females (15%). While self-rated health satisfaction was comparable, more females reported dissatisfaction. Males were more likely to work in government, institutions, and companies. They also had higher odds of being married, having pension and medical insurance, and earning more. Additionally, males predominated in smoking and alcohol consumption, and reported higher life and health satisfaction. No significant gender differences were noted in residential location, household registration, wage payment methods, or overall life satisfaction. Furthermore, we also conducted a subgroup analysis, categorizing participants into two groups based on their self-perception of health (good or not), as detailed in Supplementary material S2. These findings not only aid in our understanding of the role of gender in health inequality but also provide crucial insights for developing targeted health intervention measures.

### Multivariate analysis of the self-rated health of the male and female respondents

**Figure 2:**
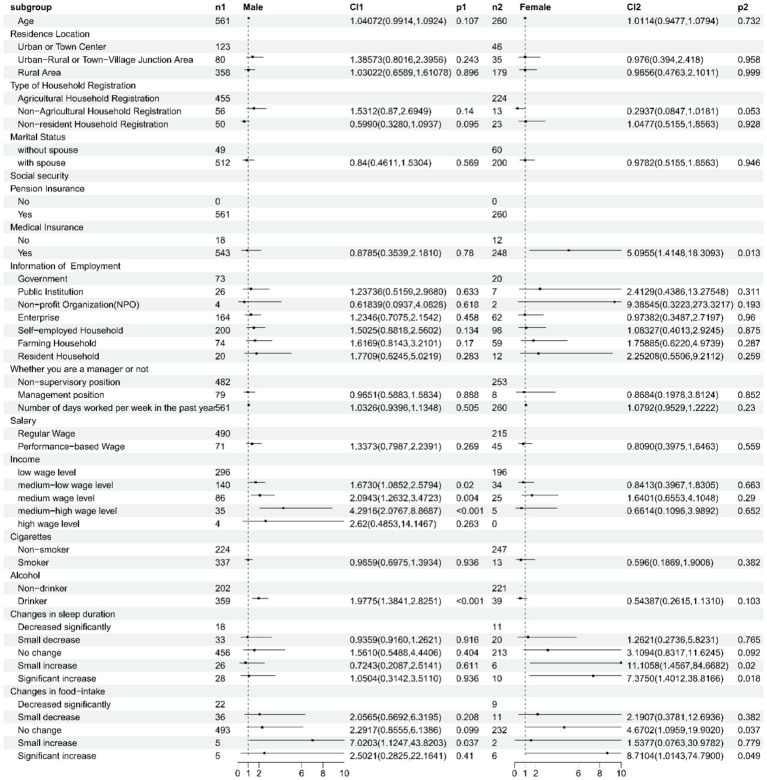
Gender-specific determinants of self-rated health among male and female older adult workers in China.

Figure 2 illustrates the results of a multivariate analysis focusing on gender-specific factors influencing self-rated health. Supplementary material S3 presents a detailed overview of these findings, highlighting the quantitative and qualitative aspects of gender disparities in self-rated health. The comprehensive analysis of our study reveals significant gender-specific factors influencing self-rated health, with both quantitative and qualitative aspects considered. Among males, economic indicators were paramount, particularly income level. Quantitatively, males with moderate-low (OR = 1.6730, 95% CI: 1.0852–2.5794, *p* = 0.020), moderate (OR = 2.0943, 95% CI: 1.2632–3.4723, *p* = 0.004), and moderate-high (OR = 4.2916, 95% CI: 2.0767–8.8687, *p* < 0.001) income levels exhibited significantly higher odds of better self-rated health compared to those with low income levels. Qualitatively, this suggests that economic stability plays a critical role in male self-perceived well-being. Additionally, alcohol consumption among males (OR = 1.9775, 95% CI: 1.3841–2.8251, *p* < 0.001) was positively associated with self-rated health, indicating a potential beneficial effect of moderate alcohol intake on male well-being.

For females, however, healthcare coverage and lifestyle factors emerged as significant predictors. Quantitatively, females with medical insurance coverage showed significantly better self-rated health (OR = 5.0955, 95% CI: 1.4181–10.0000, *p* = 0.013), underscoring the importance of accessible healthcare for female well-being. Furthermore, positive changes in sleep duration and food intake among females were associated with improved self-rated health, indicating the beneficial impact of healthy lifestyle practices. Specifically, females reporting no change in sleep duration (OR not reported but positively correlated) and no change in food intake (OR = 2.2917, 95% CI: 0.8555–6.1386, *p* = 0.099 for trend) had better self-rated health compared to those experiencing significant decreases. Collectively, these findings highlight the need for gender-specific health policies and interventions that target the distinct quantitative factors influencing self-rated health among males and females, while also considering their qualitative implications for overall well-being.

## Discussion

This study presents an empirical analysis of self-rated health among the older adult labor force in China, revealing significant gender differences in multiple dimensions. Univariate analysis provides rich background information for understanding these disparities, while multivariate regression analysis further elucidates the specific impacts of various factors on self-rated health for men and women. A detailed discussion of these findings follows.

### Impact of demographic characteristics

There appears to be a positive correlation between age and self-rated health, although it is not statistically significant (*p* = 0.1070). This may suggest that as people age, their perception of health is influenced by various complex factors, including chronic diseases, physical functioning, and changes in social roles (26). Furthermore, marital status significantly affects self-rated health for both men and women, with married men tending to assess their health as good compared to unmarried men, while the opposite is true for unmarried women. This could be attributed to the social support, emotional solace, and coping abilities that marriage brings. Previous research (27) has also found that marriage indirectly influences health outcomes through emotions and lifestyle habits. Therefore, for the older adult labor force, a good marital relationship is crucial for health.

### Social security and self-rated health

This study found that nearly all participants have access to pension and medical insurance, which to some extent safeguards their basic living and health needs. However, it is worth noting that although the coverage of medical insurance is high, its positive impact on women’s self-rated health is more significant (OR = 5.0955, *p* = 0.013). This may be related to higher healthcare needs and expectations among women, and it could also reflect the greater responsibility women have in caring for family health within the context of gender role division. Research has shown (28) that older adult women in China have lower coverage under the basic medical insurance for urban employees, based on the labor market situation. Overall, women exhibit a more proactive demand for health beliefs and attention to medical insurance, preventive care, and regular check-ups compared to men (29).

### The impact of working conditions on self-rated health

Regarding working conditions, this study found that the proportion of men working in non-rural areas such as government and corporate sectors is higher than that of women, while women have a higher proportion of employment in rural and remote areas. This difference is particularly significant among men (*p* = 0.045) and may be attributed to factors such as the nature of work, work intensity, and income levels in different industries. Furthermore, the proportion of men holding managerial positions is significantly higher than that of women (*p* = 0.001), which could be related to the manifestation of gender inequality in the occupational domain. The impact of income levels on self-rated health is particularly significant for men, as men with middle to high-income levels are more inclined to assess their health as good. This may reflect the importance of economic stability in men’s perception of their own health. Research has found (30) that in relatively unequal societies, people generally perceive their health to be worse. Additionally, a study (31) has also found that income inequality significantly exacerbates subjective health disparities among older adult individuals in China, especially those with low incomes. Therefore, targeted efforts should be made to improve the health and well-being of the older adult population.

### Lifestyle habits and self-rated health

Lifestyle habits have a significant impact on the self-rated health of both men and women. Specifically, consumption of cigarettes and alcohol is more prevalent among men (*p* < 0.001), and moderate alcohol consumption is positively correlated with men’s self-rated health (OR = 1.9775, *p* < 0.001). It should be noted that it may reflect the potential benefits of moderate alcohol intake or the consumption of alcoholic beverages on health (32). In contrast, women demonstrate a greater sensitivity to changes in sleep and dietary patterns. Specifically, the stability of sleep duration and dietary structure shows a significant positive correlation with women’s self-rated health. This finding highlights the critical role of healthy lifestyle habits in women’s health maintenance. Maintaining good sleep quality and a balanced dietary structure are of undeniable importance in promoting overall health among women. Therefore, encouraging women to pay attention to and improve their sleep and dietary habits would contribute to enhancing their quality of life and effectively preventing the occurrence of various health issues.

### Gender-specific factors and self-rated health

An important finding of this study is the crucial role of gender-specific factors in self-rated health assessment. Economic factors and employment status have a more pronounced impact on men’s self-rated health, while social security and lifestyle habits have a more pivotal influence on women’s self-rated health. This finding emphasizes the importance of developing gender-specific health policies and interventions. For men, strengthening economic support and career development opportunities can enhance their perception of self-rated health. For women, enhancing the social security system and promoting the adoption of healthy lifestyle habits may be beneficial.

### Limitations

Despite uncovering the significant role of gender-specific factors in self-rated health assessment, there are several limitations to this study. Firstly, the data used in this study are cross-sectional, which prevents establishing causal relationships. Future research can employ longitudinal designs to further explore the dynamic relationships among these factors. Secondly, this study did not consider other potential confounding factors such as genetics and psychological health status. Future research could control for these confounding factors to improve the accuracy of the results. Thirdly, the study sample primarily consists of a specific region or population, which may introduce certain regional and cultural differences. Future research can expand the sample size to enhance the generalizability of the findings. The fourth limitation of this study is the lack of direct comparison with international data on older workforce health, which could provide valuable insights into how the findings and implications of this research in China align or diverge with experiences in other countries. Lastly, the study’s reliance on single-year data limits its capacity to track longitudinal trends critical for comprehending temporal shifts in health outcomes. Additionally, the use of t-tests to evaluate gender disparities may fail to account for biases adequately. Future research should employ advanced statistical methods to establish causal relationships and mitigate omitted variable bias.

## Conclusion

In conclusion, the study reveals significant gender disparities among China’s older adult labor force in terms of occupation, marital status, insurance, income, health behaviors, and subjective well-being. Males are more likely to work in government, public institutions, and companies, indicating a gender imbalance in job distribution. The proportion of males with spouses is significantly higher, suggesting differences in marital status between genders. Males exhibit higher proportions of having pension insurance and medical insurance compared to females. Income disparities are observed, with higher proportions of males in low, medium, and medium-high income. Males also demonstrate higher rates of consuming cigarettes and alcohol. Moreover, males report higher levels of self-rated health compared to females. The findings highlight the need for targeted interventions to address these disparities and improve public health outcomes. For males, improving income levels and regulating and educating about consuming alcohol may enhance their self-rated health level. For females, promoting healthcare insurance coverage and encouraging healthy sleep duration and dietary habits may contribute to improving their self-rated health status.

## Data Availability

The original contributions presented in the study are included in the article/Supplementary material, further inquiries can be directed to the corresponding author.

## Footnotes

^1^ http://charls.pku.edu.cn/
